# Molecular Evolution of Apolipoprotein Multigene Family and the Original Functional Properties of Serum Apolipoprotein (LAL2) in *Lampetra japonica*

**DOI:** 10.3389/fimmu.2020.01751

**Published:** 2020-08-11

**Authors:** Qing Han, Yinglun Han, Hongyan Wen, Yue Pang, Qingwei Li

**Affiliations:** ^1^College of Life Sciences, Liaoning Normal University, Dalian, China; ^2^Lamprey Research Center, Liaoning Normal University, Dalian, China; ^3^Collaborative Innovation Center of Seafood Deep Processing, Dalian Polytechnic University, Dalian, China

**Keywords:** apolipoprotein, LAL2, lamprey, antibacterial, immune system

## Abstract

Apolipoprotein (APO) genes represent a large family of genes encoding various binding proteins associated with plasma lipid transport. Due to the long divergence history, it remains to be confirmed whether these genes evolved from a common ancestor through gene duplication and original function, and how this evolution occurred. In this study, based on the phylogenetic tree, sequence alignment, motifs, and evolutionary analysis of gene synteny and collinearity, APOA, APOC, and APOE in higher vertebrates may have a common ancestor, lamprey serum apolipoprotein LAL1 or LAL2, which traces back to 360 million years ago. Moreover, the results of immunofluorescence, immunohistochemistry, and flow cytometry show that LAL2 is primarily distributed in the liver, kidney, and blood leukocytes of lampreys, and specifically localized in the cytoplasm of liver cells and leukocytes, as well as secreted into sera. Surface plasmon resonance technology demonstrates that LAL2 colocalizes to breast adenocarcinoma cells (MCF-7) or chronic myeloid leukemia cells (K562) associated with lamprey immune protein (LIP) and further enhances the killing effect of LIP on tumor cells. In addition, using quantitative real-time PCR (Q-PCR) and western blot methods, we found that the relative mRNA and protein expression of *lal2* in lamprey leukocytes and sera increased significantly at different times after stimulating with *Staphylococcus aureus, Vibrio anguillarum*, and Polyinosinic-polycytidylic acid (Poly I:C). Moreover, LAL2 was found to recognize and bind to gram-positive bacteria (*Staphylococcus aureus* and *Bacillus cereus*) and gram-negative bacteria (*Escherichia coli*) and play an important role in the antibacterial process. All in all, our data reveals a long, complex evolutionary history for apolipoprotein genes under different selection pressures, confirms the immune effect of LAL2 in lamprey sera against pathogens, and lays the foundation for further research regarding biological functions of lamprey immune systems.

## Introduction

As early as 1987, Pontes et al. (1) founded two abundant apolipoproteins in the “high-density lipoprotein fraction” of ultracentrifuged plasma in *Petromyzon marinus*, designated lamprey apolipoproteins LAL1 and LAL2. Their amino acid compositions were similar to portions of apolipoprotein A-IV sequence in mammalian blood. However, the existing database indicates that LAL2 has no apolipoproteinA/E or C domain. Their functions are not fully elucidated, and it remains elusive whether they are the ancestors of vertebrate apolipoproteins. However, it is well-known that apolipoprotein is a protein component of plasma lipoproteins (2, 3). Apolipoproteins of vertebrates are primarily synthesized in the liver and small intestine, which are involved in the transport and redistribution of lipids between different tissues and cells through blood (4, 5). Research shows that several apolipoproteins also play important roles in antibacterial and antiviral processes (6–9). For example, APOA1-containing high-density lipoprotein particles (HDL) exert antibacterial activity by directly affecting the growth of bacteria and promoting the self-defense mechanism of normal and immunocompromised individuals (10). This function is attributed primarily to the ability of APOA1 to bind and neutralize both bacterial endotoxin lipopolysachharide (LPS) and lipoteichoic acid (LTA) (11–14). Moreover, whether it is expressed *in vivo* or injected, APOA1 elictis an antiviral effect on enveloped and non-enveloped DNA and RNA viruses by directly causing viral inactivation. Specifically, APOA1 has been shown to arrest virus-induced cell fusion in the blood during human immunodeficiency virus (HIV) and Herpes virus infection, thereby preventing the virus from penetrating into the cell (15). Additionally, highly conserved alternating cationic/hydrophobic motifs have been identified in the APOC1 sequence that participate in binding to LPS and enhanced biological response to LPS via a mechanism similar to lipopolysaccharide-binding protein (LBP) (16). Meanwhile, APOL-1 has pore-forming microbicidal activity that can cause lysis and death of trypanosome (17).

The lamprey is a member of an ancient lineage of jawless fish that stem ~550 million years ago and has served as a crucial model for understanding conserved features that are relevant to biomedicine. Lampreys have adaptive immune systems with variable lymphocyte receptors (VLRs) and innate immune systems with complement related molecules to prevent the invasion of various foreign pathogens, such as mannose binding lectin (MBL), complement C1q, C3, etc. (18–20). Lamprey immune protein (LIP), a cytotoxic protein, has a jacalin-like domain and an aerolysin pore-forming domain previously identified in granulocytes of *Lampetra japonica* (21). We demonstrate the crystal structure of LIP and the mode of action involving dual selective recognition and efficient binding dependent on both N-linked glycans on GPI-anchored proteins (GPI-APs) and sphingomyelin (SM) in lipid rafts (22). LIP can kill a panel of human cancer cells yet has minimal effects on normal cells. MCF-7 and K562 cells stimulated with LIP exhibited the generation of chemokines and proinflammatory molecules, and increased the expression of genes in the calcium signaling pathway, ROS signaling pathway, and natural killer cell-mediated cytotoxicity pathways (23, 24). However, it remains unclear whether large amounts of LAL2 in serum interacts with LIP molecule and participates in the immune response.

In the present work, we elucidated the molecular evolution process of LAL2 and LAL1 and determined their relationship with vertebrate orthologs and paralogs. We further investigated LAL2 expression patterns in gill, supraneural body, heart, liver, intestine, and kidney, and also intracellular localization in liver cells and leukocytes. Simultaneously, the potential interaction between LAL2 and LIP was verified, and the addition of LAL2 was found to enhance the killing activity of LIP in lamprey. Moreover, the antibacterial and antiviral activities of LAL2 were examined to shed light on its role in immunity. Exploring the biological function of LAL2 lays the foundation for clarifying antibacterial function in lamprey and provides a reference for the research of innate immune mechanisms of lamprey.

## Materials and Methods

### Animals and Cell Culture

Adult *L. japonica* (length: 36–42 cm, weight: 75–112 g) and *Lampetra morii* (length: 20–25 cm, weight: 18–23 g) were obtained from the Songhua River from Heilongjiang Province, China. The lampreys were housed in fully automatic water purification tanks at 4–6°C. All animals were in good condition before the experiments.

MCF-7 cells and K562 cells, purchased from the American Type Culture Collection (Manassas, VA) were maintained in RPMI 1640 medium (Sigma-Aldrich, USA) supplemented with 10% fetal bovine serum (Sigma-Aldrich, USA), 100 U/mL penicillin (Sigma-Aldrich, USA), and 100 mg/mL streptomycin (Sigma-Aldrich, USA). Cells were cultured in an incubator humidified with 5% CO_2_ and 95% air at 37°C.

*S. aureus, B. cereus, Vibrio anguillarum*, and *E. coli* strains were isolated from the intestine of the lamprey. *S. aureus* (28°C), *B. cereus* (28°C), and *E. coli* (37°C) strains were cultured in Luria broth liquid medium with 1% peptone, 1% NaCl, and 0.5% yeast extract (Sangon Biotech, Shanghai, China). The *V. anguillarum* (28°C) strain was cultured in 2216E liquid medium with 0.5% peptone, 0.1% yeast extract, and seawater (pH = 8.0). All the strains were supplied by College of Life Science, Liaoning Normal University (Dalian, China).

### Sequence Analysis, Sequence Alignments, and Phylogenetic Analysis

The amino acid sequences of lamprey apolipoprotein LAL1 and LAL2 were obtained from the *L. japonica* three-generation sequencing library and *Lethenteron reissneri* database from our laboratory. The amino acid sequences of the corresponding apolipoprotein family genes in other species are from NCBI (https://www.ncbi.nlm.nih.gov/) and Ensembl (http://asia.ensembl.org/index.html) database for sequence alignment by Bioedit 7.0. Two comparisons of syntenic genomic regions, respectively containing *lal1* and *lal2* genes, were completed using *L. reissneri* databases and the Genomicus website (http://www.genomicus.biologie.ens.fr/genomicus-92.01/cgi-bin/search.pl). Thereafter, a phylogenetic tree was constructed using the neighbor-joining (NJ) method using MEGA 7.0 software and the bootstrap test (1,000 replicates). The tree was drawn to scale, with branch lengths in the same units as those of the evolutionary distances used to infer the phylogenetic tree. Functional domains of the apolipoprotein family genes were analyzed using the NCBI website (https://www.ncbi.nlm.nih.gov/Structure/cdd/wrpsb.cgi). Motif analysis was performed using the MEME website (http://meme-suite.org/) and TB (Toolbox for Biologists) tools software.

### Expression of Recombinant LAL2 (rLAL2) Protein and Preparation of Antibodies

The prokaryotic expression vector pET32a-*lal2* was constructed to obtain the recombinant LAL2 (rLAL2) protein as described previously (25). Rabbit anti-LAL2 polyclonal antibody was generated through subcutaneous injection of New Zealand white rabbits with purified rLAL2 protein over 8 weeks, as described previously (26). The antibody titer in the anti-rLAL2 serum was determined via enzyme-linked immunosorbent assay (ELISA) at different dilutions, and the titer increased 640,000-fold over pre-immunization levels (pre-immunized rabbit IgG was used as a negative control). The antibody specificity was confirmed using western blot assays; rLAL2 and lamprey serum were subjected to 12% SDS-PAGE and transferred onto nitrocellulose membranes (Invitrogen, USA). The membranes were blocked with 5% non-fat powdered milk (Sangon Biotech, Shanghai, China) and incubated with rabbit anti-LAL2 (1 μg/mL) antibody overnight at 4°C, followed by incubation with 1.2 μg/mL HRP-conjugated goat anti-rabbit IgG (Sangon Biotech, Shanghai, China). Next, membranes were washed four times with tris-buffered saline Tween-20 (Sangon Biotech, Shanghai, China) and developed with enhanced chemiluminescence (ECL) substrate (Tanon, China) using Alpha FluorChem®Q (Cell Biosciences, USA).

### Purification of Natural LAL2 Protein With Anion Exchange Chromatography

Serum from *L. japonica* was dialyzed in buffer A consisting of 20 mM KPB, 0.1 M KCl and 5% glycerol, at pH 7.0 at 4°C. The dialyzed fraction was filtered through a 0.22 μM membrane and was applied to a 10 mL × 2 MacroPrep Ceramic Hydroxyapatite column equilibrated with buffer A. The column was then washed with the same buffer and eluted with a linear gradient from 0 to 250 mM KPB in buffer A. The pooled fractions containing protein activity from the above column were dialyzed in buffer B consisting of 20 mM Tris-HCl and 5% glycerol, at pH 8.0 at 4°C. The dialyzed fraction was applied to a 20 mL Q Sepharose Fast Flow column equilibrated with buffer B. After washing, the sample was eluted with a linear gradient from 0 to 300 mM of KCl in buffer B. During the separation and purification of the active components of lamprey serum (TaKaRa, Dalian, China), we observed an abundance of natural LAL2 protein in the eluted sample No. 46. It was diluted with low-salt buffer (20 mM Tris-HCl, pH = 9.0), passed through the anion exchange column (HiTrap Q HP_1 mL, General Electric Company, USA) at a rate of 0.8 mL/min using ÄKTA pure (General Electric Company, USA), and impure proteins were removed with low-salt buffer at a rate of 1 mL/min. High-salt buffer (20 mM Tris-HCl, 1 M NaCl, pH = 9.0) was gradually added to the low-salt buffer, finally the target protein was eluted at a rate of 1 mL/min using mixed buffer in a time-dose dependent manner. After detection using 15% SDS-PAGE, it was dialyzed using PBS (145.3 mM NaCl, 8.4 mM Na_2_HPO_4_, and 2 mM NaH_2_PO_4_). All protein concentrations were measured using the Bicinchoninic acid (BCA) protein assay kit (Sangon biotech, Shanghai, China).

### Immunofluorescence and Flow Cytometry Were Used to Detect the Localization of LAL2 and LIP

Lamprey liver cells and blood leukocytes were isolated, fixed, permeabilized, and blocked with fetal bovine serum as previously described (27). Briefly, cells were incubated with rabbit anti-rLAL2 antibody (0.8 μg/mL) at 4°C overnight. The next day, cells were washed twice with PBS (140 mM NaCl, 2.7 mM KCl, 10 mM Na_2_HPO_4_, and 1.8 mM KH_2_PO_4_) and incubated with Alexa Fluor 488-labeled donkey anti-rabbit IgG (Thermo Fisher, USA). Following washing with PBS, the cells were stained with 4′,6-diamidino-2-phenylindole (DAPI). After washing twice with PBS, the cover slips were mounted on glass slides with one drop of antifade solution. The immunofluorescence was visualized and captured using LSM 710 Laser Scanning Confocal Microscopy (Carl Zeiss Inc, Germany) and analyzed using Zeiss ZENLE software. The lamprey liver cells and leukocytes were fixed for 15 min in 4% paraformaldehyde with PBS at room temperature. Thereafter, cells were treated according to the method described above to detect the expression of LAL2 in liver cells and leukocytes using flow cytometry (BD Biosciences, USA). The flow cytometer was set at 488 nm (excitation wavelengths) to detect green fluorescence. Cells incubated with normal rabbit IgG were used as isotype controls.

In the same way, MCF-7/K562 cells were observed for 2 h with rLAL2 protein and rLAL2 + LIP protein incubation. When the rLAL2 + LIP incubated group exhibited bubbling, all cells were fixed, blocked using fetal bovine serum, incubated with rabbit anti-rLAL2 antibody (primary antibody), followed by Alexa Fluor 555-labeled donkey anti-rabbit IgG antibody (second antibody) to detect the localization of LAL2 protein on MCF-7/K562 cells. After labeling LIP protein with Alexa Fluor 488 (Microscale Protein Labeling Kit, Invitrogen, USA), four experimental groups: Alexa 488-LIP, rLAL2-1 μg + Alexa 488-LIP, rLAL2-2 μg + Alexa 488-LIP, and rLAL2-3 μg + Alexa 488-LIP, were established to detect the location of LIP protein on the MCF-7/K562 cells. The cells were analyzed on a FACSAria flow cytometer (BD Biosciences, USA), which was set at 488 and 555 nm (excitation wavelengths) to detect green and red fluorescence, respectively.

### The Synergistic Effect of rLAL2 on LIP Killing

MCF-7 cells (cultured in 96 well plate) and LIP protein were incubated with rLAL2 for 3 h. According to the experimental sequence in Figure 4D, MCF-7 cells were incubated with PBS, 2 μg LIP, 2.5 μg rLAL2, or 5 μg rLAL2, LIP was added to the MCF-7 cells that pre-incubated with 2.5 μg or 5 μg rLAL2, 2.5 μg/5 μg rLAL2, and LIP were pre-incubated and then the mixture was added to MCF-7 cells. Thereafter, cells were stained for 5 min using Hoechst (blue, Beyotime, Shanghai, China) and propidium iodide (PI, red, Thermo fisher, USA), finally analyzed on the Operetta™ High-content machine (PerkinElmer, USA).

MCF-7 and K562 cells were cultured and collected, the cells were divided into five groups with the addition of PBS, 5 μg rLAL2, 2 μg LIP, 2.5 μg rLAL2 + 2 μg LIP, and 5 μg rLAL2 + 2 μg LIP as shown in Figure 4F, respectively. Followed by staining with Hoechst and PI for 5 min. Cells were then filtered and analyzed using a flow cytometer set at 560 nm(excitation wavelength), and analyses were performed by using Cell Quest Pro software. The appropriate FSC voltage and threshold were adjusted, inspector-gate, G1 = R1, to regulate the fluorescence voltage to set the negative control and the compensation between the fluorescence. The samples were then loaded in order, and the data files were obtained.

### Surface Plasmon Resonance Analysis

LIP or rLAL2 protein were coupled on the second channel of a CM5 chip using buffer (pH = 4.0), while the first channel was used as a reference channel, both chips were activated with NHS/EDC and blocked with ethanolamine. LIP or rLAL2 in HBS-EP solution was flowed through the rLAL2 or LIP chip. The analyte, rLAL2 or LIP protein, was diluted in the same buffer. The Biacore T200 (General Electric Company, USA) was used for the experiment and subsequent analysis. TRX was used as a negative control, using the same experimental method.

### Quantitative Real-Time PCR (Q-PCR)

Adult *L. japonica* were divided into three groups (three animals per group), each immunized with 100 μL *S. aureus, V. anguillarum* (suspended to 1 × 10^8^ cells/mL in normal saline), or Poly I:C (0.1 mg/mL) for 0 h (immediately following addition of stimulus), 2, 8, 24, 48, and 72 h via intraperitoneal injections, respectively. Adult lamprey blood was collected by cutting the tail, and leukocytes were isolated from blood by Ficoll-Paque gradient centrifugation with lymphocyte separation solution (160 × *g*, 20 min) (TBD, China). *L. morii* were immunized using the same method to collect sera. The gill, supraneural body, heart, liver, intestine, kidney, and leukocytes were obtained from normal adult *L. japonica*. Total RNA was extracted from the tissues and cells using Trizol (Invitrogen, USA), and the RNA was treated with DNase I (TaKaRa, Dalian, China). Reverse transcription that each group of RNA is quantified to 2 μg was performed using gDNA Eraser (PrimeScript™ RT reagent Kit) as described by the manufacturer (TaKaRa, Dalian, China). Real-time quantitative PCR was conducted using a SYBR® PrimeScript™ RT-PCR Kit (TaKaRa, Dalian, China) according to the manufacturer's protocol. The PCR was performed in a 25 μL volume, consisting of 2 μL cDNA (diluting to 50 ng/μL), 12.5 μL SYBR Premix Ex Taq, 1 μL of each primer (10 μM), and ddH_2_O. The gene expression in each sample was normalized relative to the *gapdh* gene (GenBank accession no. KU041137.1). The reaction efficiency was tested by gradual dilution of the cDNA template (1, 5, 10, 20, and 40×). The amplification efficiency of all primers was confirmed to be between 0.9 and 1.1, and the specificity of the amplification reaction was analyzed by dissociation curve analyses. The primer sequences used are as follows: *lal2*-F: 5′-ACGGTCCACCTGCACGAAT-3′; *lal2*-R: 5′-TTCACCTCCTTCATCAGTCCAA-3′. L-*gapdh*-F: 5′-AACCAACTGCCTGGCTCCT-3′; L-*gapdh*-R: 5′-GTCTTCTGCGTTGCCGTGT-3′.

### Scanning Electron Microscopy (SEM) for Bacterial Morphology

The bacteria were incubated with PBS and 5 μM LAL2 at 4°C for 12 h, fixed with 2.5% glutaraldehyde (Kemiou, China) at 4°C overnight, and dehydrated at various ethanol gradients: 30, 50, 80, and 100%. In 100% ethanol, point samples on tables were sprayed with gold and photographed using scanning electron microscopy.

### Enzyme Linked Immunosorbent Assay (ELISA) Analysis of the Interaction Between LAL2 Protein and Microbial Components

Plates were coated with various microbial components (0.2 μg/well) at 4°C overnight, then were washed and incubated with different concentrations of LAL2 (0, 10, 20, 50, 100, 200 nM) at 37°C for 3 h, 1% BSA was added, followed by detection with 100 μL/well rabbit anti-LAL2 antibody (4 μg/mL) and goat anti-rabbit antibody (1.5 μg/mL). ELISA substrate (100 μL/well, Solarbio, USA) was added and incubated for 15 min at 37°C, color development was halted through the addition of 2 M H_2_SO_4_ (50 μL/well). The plates were washed thrice with PBST (PBS with 0.05% Tween-20) between steps. One representative experiment of three is shown. Background absorbance without LAL2 protein and with anti-LAL2 antibody was subtracted as a negative control (NC).

### Statistical Analysis

All calculations were performed using GraphPad Prism 7 (GraphPad Software Inc, USA). The data are presented as the mean ± S.E. The significance of the differences between the mean values was determined using Microsoft Excel 2007. In all cases, ^*^P < 0.05 was considered a statistically significant difference, ^**^P < 0.01 was considered a very significant difference, and ^***^P < 0.001 was considered an extremely significant difference.

## Results

### Identification and Purification of Lamprey LAL2

**Figure 1:**
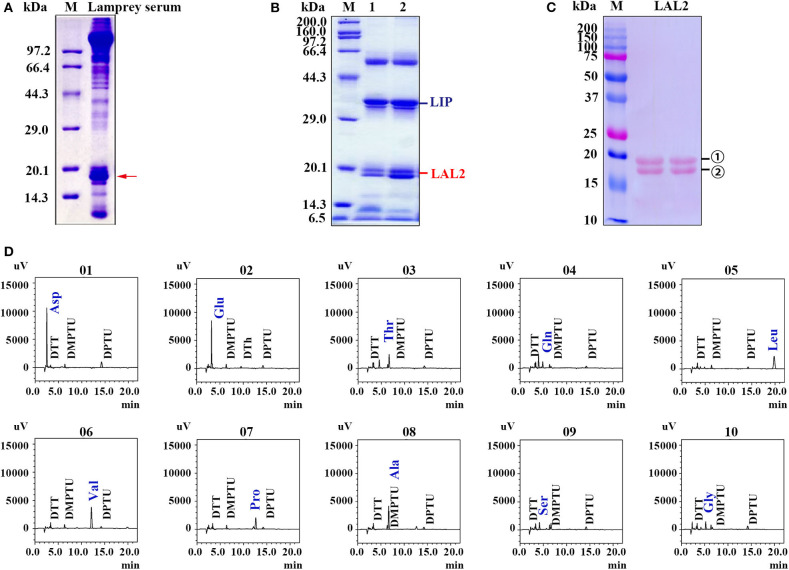
Isolation and identification of LAL2 from lamprey serum. **(A)** Electrophoretic patterns of lamprey serum proteins via 15% SDS-PAGE. M, low molecular-weight protein marker. Red arrow points to apolipoprotein LAL2. **(B)** Detection of killer components in lamprey sera via 12% SDS-PAGE. **(C)** Electrotransfer atlas of LAL2 from lamprey serum that dyed with Ponceau S. M, Dual marker (Bio-Rad), bands for number 1 and 2 both represent LAL2. **(D)** Ten amino acid test maps of LAL2 N-terminal sequence (number 1).

In our previous study, lamprey sera exerted important cytotoxic effects on tumor cells (28). This is evidenced by the morphological changes and cell organelle damage observed in cervical cancer cells (HeLa) and acute promyelocytic leukemia cells (NB4) treated with lamprey serum during a 15 min incubation (28). To identify this cytotoxic protein in the sera (Figure 1A), lamprey sera was purified using a hydroxyapatite column and a Q Sepharose Fast Flow column. The fraction of protein activity was determined by the degree of cell membrane disruption. When these fractions with active protein were collected and analyzed via 12% SDS-PAGE, a protein band was observed at ~34 kDa molecular-weight (Figure 1B). According to the liquid chromatography-tandem mass spectrometry (LC/MS/MS) analysis of tryptic-digested peptides, the purified protein was identified as LIP. In addition, we observed two protein bands positioned at ~15–20 kDa. The two proteins were identified as LAL2 by mass spectrometry (Tables S1, S2). To detect the difference between the two LAL2 proteins (in Figure 1C), N-terminal sequencing was performed by Edman degradation and online analysis of High-Performance Liquid Chromatography at the Shanghai Life Science Research Institute. As shown in Figure 1D and Figure S1, the first ten amino acids of the two bands were identical (NH2-Asp-Glu-Thr-Gln-Leu-Val-Pro-Ala-Ser-Gly), which may be the result of glycosylation. Of course, it may also be possible that the C-terminal peptide of LAL2 was degraded. The open reading frame (ORF) of LAL2 has 576 bp and encodes a total of 191 amino acid residues. The first 23 amino acids coded after the initiation ATG are characteristic of a signal peptide.

### Evolutionary Analyses of LAL1 and LAL2

**Figure 2:**
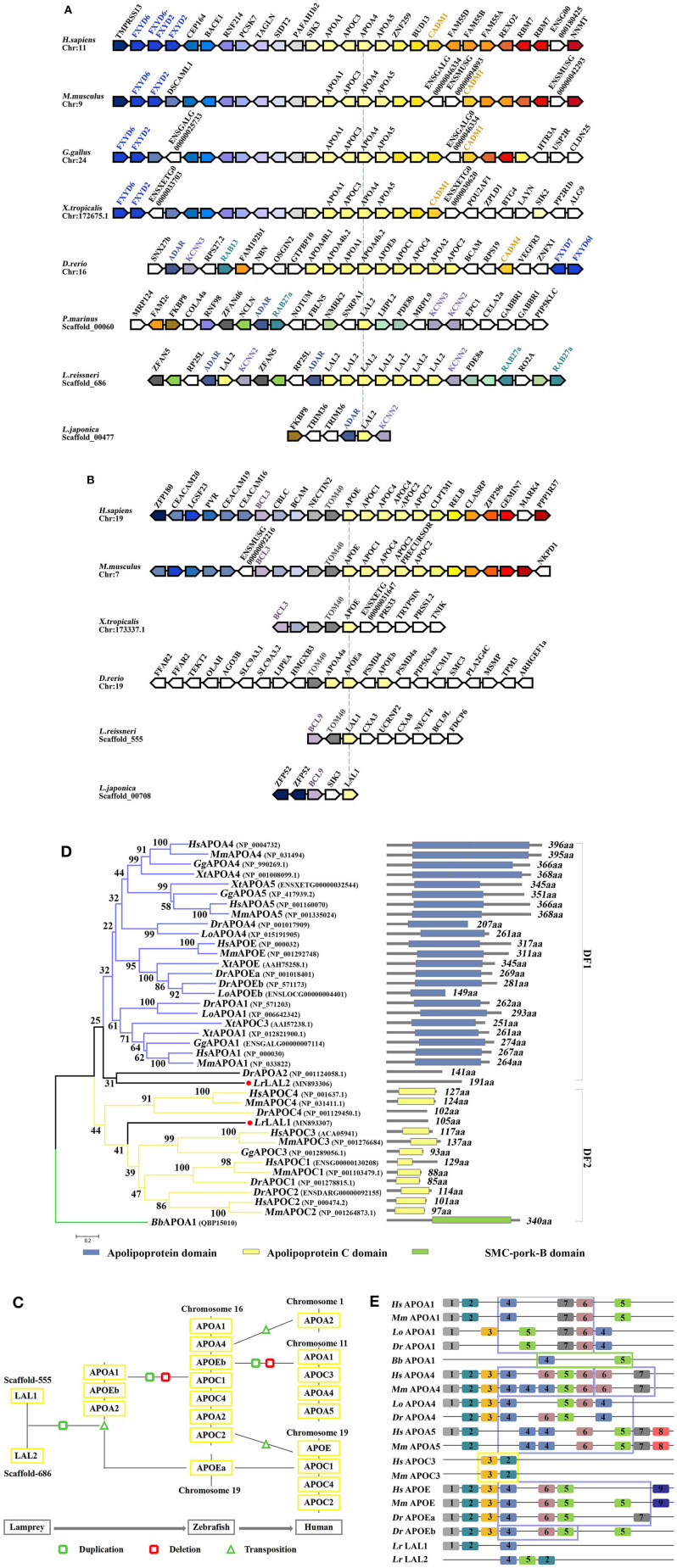
Evolutionary analyses of LAL2. **(A,B)** The conservation of genes neighboring apolipoprotein family genes, the arrowheads pointing in opposite directions indicate genes located on opposite strands. Chr denotes the chromosome. **(C)** Model diagram of vertebrate apolipoprotein evolution. **(D)** The phylogenetic tree was constructed based on the 39 full-length protein sequences of APOA, APOC, APOE, LAL1, and LAL2. The optimal tree with the sum of branch length = 12.09372528 is shown and the percentage of replicate trees in which the associated taxa clustered together in the bootstrap test are shown next to the branches. The section on the right indicates the corresponding domain composition of the sequence in left. **(E)** A motif composition of the apolipoprotein family. The motifs, which are numbered 1–9, are shown in different colored blocks. The regions fenced by blue lines represent “apolipoprotein” domain, similarly the yellow represent “APO-CIII” domain, the green represents “SMC-pork-B” domain.

The LAL2 proteins were purified and identified in the “high-density lipoprotein fraction” of plasma from *Petromyzon marinus* (1). However, high homology in amino acid sequences was not observed between LAL2 and the apolipoproteins of other species, and the domain in LAL2 were not found to be similar to mammalian blood apolipoproteins. In the current study, the amino acid sequence alignment results of LAL2 revealed that LAL2 (*Lampetra japonica*) display more than 90% sequence similarity to blood plasma LAL2 from *Petromyzon marinus, Lampetra fluviatilis*, and *Lethenteron reissneri* (Figure S2). To better understand the evolution of the *lal2* gene family during the vertebrate evolutionary process, the neighboring gene environment of lamprey *lal2* was compared among fish, amphibian, bird, and mammals. Using the draft genome assemblies of *Lethenteron reissneri*, we were able to assign orthology of the lamprey genes based on conserved synteny for genes directly surrounding the *lal2* gene (Figure 2A). A comparison of genomic regions containing *lal2* genes shows that there are eight *lal2* loci on the *L. reissneri* scaffold_686, no introns, and similar gene groups as sea lamprey in its surrounding. Strong syntenic relationships among LAL2 gene orthologs were easily detected in three jawless vertebrates (*L. reissneri, P. marinus*, and *L. japonica*) genome sequences that we examined. Three genes (*adar, kcnn*, and *rab)* surrounding *lal2* are also found in the neighborhood of zebrafish *apoa1, apoa2, apoa4, apoc1, apoc2, apoc4*, and *apoe*. In addition, this analysis confirmed that orthologs and paralogs of mammalian *apo* are present in birds, amphibians, and bony fishes. Near to the *lal2* gene in the lamprey, the *fxyd* and *cadm* genes, although undetectable, could be examined for syntenic relationship with *apo* neighborhood in other vertebrates. Unfortunately, the tandem LAL2 sequences were unidentified based on the current sea lamprey genome data, thus, it is not possible to define the quantity of LAL2 on the chromosome of sea lamprey. And the adjacent genes of LAL2 are unable to determine perfectly because of poor sequencing and splicing results in *Lampetra japonica* genome. Furthermore, we also found *lal1* and *tom40* to be evolutionarily linked, and located on the *L. reissneri* scaffold_555 (Figure 2B). And *lal1* is always accompanied by *bcl* in lamprey, zebrafish, frog, mouse, and human, the chromosome on which it is located is relatively stable. However, we only find *lal1* in scaffold_05301 with no neighboring gene environment from *Petromyzon marinus* Germline Genome. And there is no *apoe* genomic information for reptiles and birds in genomics database. In a word, it is possible that close genomic proximity of *lal2* and *apoa* evolved, while LAL1 is located in close proximity to *apoe* in fish and mammals (Figure 2C), *apoc1, 2, 4* were formed by the replication and gene deletion of *apoa* and translocated to the periphery of *apoe* (29).

The phylogenetic tree based on the alignment of *Branchiostoma belcheri* APOA1, and other apolipoprotein amino acid sequences, involved in co-linearity was drawn using the NJ methods. *Lepisosteus oculatus* APOA1, APOA4, and APOEb were added to ensure the stability of the phylogenetic tree. As shown in Figure 2D, the BbAPOA1 was placed apart. The overall topology supported two main clusters, which corresponded to APOA/E and APOC vertebrate families, while lamprey LAL2 sequence was considered closer to APOA/E vertebrate family, similar to DrAPOA2 and equivalent to the outgroups of distinct family 1 (DF1). Based on the results of collinear analysis, this suggests that LAL2 is likely a common ancestor of vertebrate APOA1, A4, A5, and E. Lamprey LAL1 sequence was similarly considered closer to the APOC vertebrate family and formed distinct family 2 (DF2) with an improved bootstrap value and compact structure. Notably, the branch of LAL1 is also an outgroup equivalent to DF1. The results of phylogenetic analysis demonstrated that lamprey LAL1 and LAL2 are likely to have common ancestors, and APOA/E and APOC in the vertebrate lineage arose by duplication and reorganization of LAL2 and LAL1, respectively.

Our predicted results using the PSIPRED website show that the α-helix of the LAL2 and LAL1 secondary structures account for 78.01 and 79.05% of the secondary structure, respectively (Figures S3A,B). Furthermore, circular dichroism shows the secondary structure of LAL2, similar to APOA1, to be comprised primarily of α-helices (Figures S3C,D). To analyze conservation of the amino acid sequence, after searching select representative sequences to predict motif composition (Figure 2E, Table S3), we observed that when MEME selects nine motifs to analyze the apolipoproteins of each species, amphioxus and lamprey display 2–3 motifs, APOC3 possesses only two motifs, while other apolipoproteins of zebrafish, spotted gar, mouse, and human possess 5–10 motifs. From the types of motifs is was found that LrLAL1 has the same motif 1, 2, and 4 as zebrafish, spotted gar, mouse, and human, while motifs 2, 4, and 5 exist in LrLAL2 and apolipoprotein A and E family sequences of most jaw vertebrates. Furthermore, motifs 3, 6, and 7 evolved from fish apolipoproteins, and motifs 8 and 9 are unique to mammalian APOA5 and APOE, respectively. The apolipoprotein domain of APOA1, APOA4, and APOE from jawed vertebrates, which is associated with lipid particles and may function in lipoprotein-mediated lipid transport, is primarily composed of motifs 4, 5, 6, and 7. These proteins contain several 22 residue repeats which form a pair of α-helices. Meanwhile the APOC3 “APO-CIII apolipoprotein” domain is primarily composed of motifs 2 and 3, which inhibit lipoprotein lipase (LPL) activity and play roles in triglyceride metabolism. It is obvious that LrLAL1 and LrLAL2 retain parts of the same motif as the above-mentioned domain, however, they do not possess the integral apolipoprotein domain. Therefore, it is suggested that the apolipoprotein superfamily from lamprey LAL1 and LAL2 to mammal APOA, APOC, and APOE has acquired most of its structural and functional innovations throughout vertebrate evolution.

### The Expression Pattern of LAL2 in Lamprey Tissues and Cells

**Figure 3:**
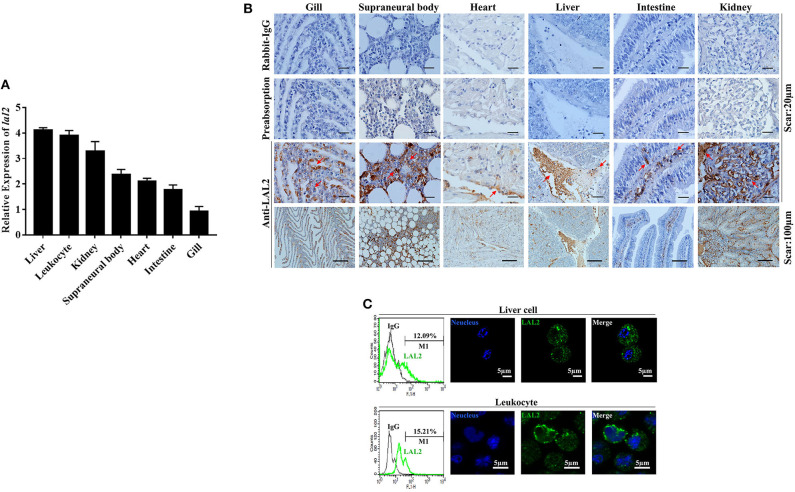
The tissue distribution and cellular localization of LAL2. **(A)** The expression level of *lal2*, which was compared with *gapdh* in various lamprey tissues using Q-PCR. **(B)** Distribution and localization of LAL2 as observed by immunohistochemical staining, in which rabbit IgG is an isotype control, LAL2 with antibody incubating group (preabsorption) was used as a negative control, the upper parts of group anti-LAL2 are the corresponding enlarged view below. **(C)** Flow cytometry and immunofluorescence was used to analyze the localization of LAL2 in lamprey liver cells and leukocytes, as compared to IgG. Images were taken by laser confocal microscopy with the fluorescent cell-labeling dye DAPI (blue) and Alexa Fluor 488 goat anti-rabbit IgG (green). Scale bar: 5 μm.

The relative expression levels of *lal2* gene in the lamprey gill, supraneural body, heart, liver, intestine, kidney, and leukocytes were detected using Q-PCR (Figure 3A). LAL2 is expressed in these tissues or cells, and the expression levels in liver, leukocytes, and kidney were relatively high. To detect the distribution of natural LAL2 protein in different tissues of the lamprey, we purified rLAL2 migrated as a single band using a 12% SDS-PAGE gel with a molecular mass of ~38 kDa (Figure S4A), and prepared LAL2 rabbit polyclonal antibody, which specifically recognized rLAL2 and native LAL2 (Figures S4B,C). The band around 40 kDa in the serum sample was identified as LAL2-dimer using LC-MS/MS analysis (Figure S4D). The localization analysis was performed using immunohistochemistry as described previously (24), and LAL2 was primarily expressed in the epithelial cells and blood cells of the gill, blood cells of the supraneural body, endothelial cell area of the heart, venous areas of the liver, epithelial cells of the intestine, and venous and epithelial cells of the kidney (Figure 3B). The expression was relatively high in several tissues, such as liver, kidney, and supraneural body. The lamprey liver cells and leukocytes were fixed separately to further detect LAL2 expression at the protein level. Furthermore, intracellular localization of LAL2 was revealed using flow cytometry. Results showed that LAL2 was expressed in both liver cells and leukocytes (Figure 3C, left), which was observed in the cytoplasm using confocal microscopy (Figure 3C, right).

### LAL2 Can Promote the Localization and Killing Effect of LIP on MCF-7 and K562 Cells

**Figure 4:**
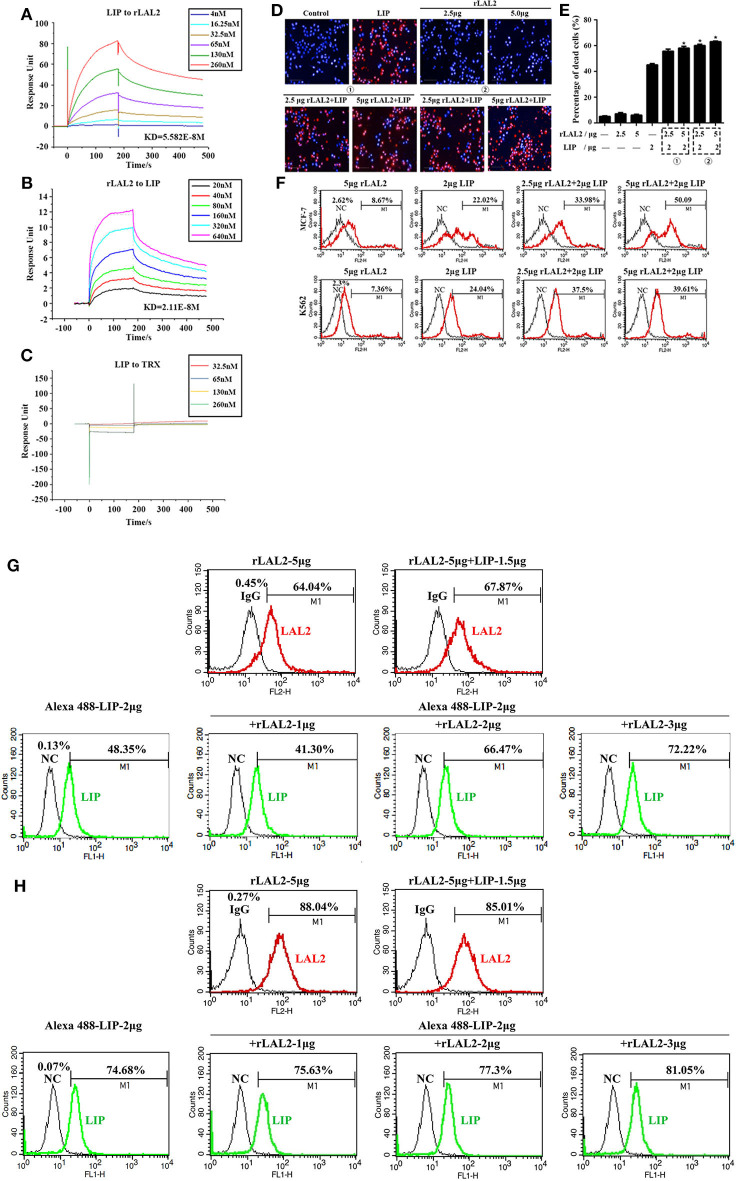
LAL2 plays a synergistic role in LIP killing the tumor cells. **(A)** Biacore analysis of the interactions between different concentrations of rLAL2 and immobilized LIP [in resonance units (RU)]. **(B)** Biacore analysis of different concentrations of LIP and immobilized rLAL2 (in RU). **(C)** Biacore analysis of different TRX concentrations and immobilized LIP. **(D,E)** High-content analyzed the killing effect of LIP on MCF-7 cells after incubation with rLAL2 (*n* = 3), scale bar: 100 μm. The statistical map is calculated from six fields randomly selected for each well. **(F)** Flow cytometry was used to analyze the killing effect of LIP on MCF-7/K562 cells after incubation with rLAL2. **(G,H)** Effect of rLAL2 on the localization of LIP molecule on MCF-7/K562 cells with the Alexa 488-LIP (green) and Alexa 555-goat anti-rabbit IgG (red).

LIP is a cytotoxic lamprey protein, which plays an important role in tumor cell survival and growth (21–24). Previously, during the process of purifying the cytotoxic protein in lamprey serum, it was found that LIP and LAL2 were always present in an eluted sample (Figure 1B). To determine whether there is a certain interaction between LIP and LAL2, rLAL2 or LIP protein were anchored to the surface of the chip using surface plasmon resonance technology, different concentrations of LIP or rLAL2 were used as analytes, and affinity kinetics fitting analysis was performed: rLAL2 protein flowed through the anchored LIP chip, the affinity KD was 5.582E-8M, LIP flowed through the anchored rLAL2 chip, and the affinity KD was 2.11E-8M. Both KD values reflect the same magnitude. This fully demonstrates the strong interaction between rLAL2 and LIP (Figures 4A,B), and suggests that LIP has no interaction with TRX (Figure 4C).

LIP exerts a specific killing effect on certain tumor cells (23, 24). The selective killing mechanism proposes that LIP could bind to biantennary bi-sialylated non-fucosylated N-glycan of cancer cells, such as MCF-7 and K562 cells, and not affect normal cells (22). It was speculated that the interaction between rLAL2 and LIP may also influence LIP killing activity, the MCF-7 cells sensitive to LIP are plated in 96-well plates for overnight culture, according to the experimental design. Results of PI/Hoechst staining and high-content analysis indicated that rLAL2 treatment alone had no impact on MCF-7 cells. However, rLAL2 significantly promoted the killing effect of LIP on MCF-7 cells in a dose-dependent manner (^*^P < 0.05, Figures 4D,E), regardless of whether rLAL2 protein was incubated with MCF-7 cells alone before LIP protein addition or a mixture of rLAL2 and LIP was added to the cells. In order to further verify the synergy of rLAL2 on the killing activity of LIP, K562 cells were analyzed, showing results identical to those of MCF-7 cells. Furthermore, the effect of rLAL2 on the killing activity of LIP was analyzed using a combination of PI staining and flow cytometry, indicating that the results are consistent with the above results (Figure 4F). In summary, our findings show that LAL2 play a major role in assisting LIP to kill tumor cells.

To investigate the localization of LIP on MCF-7 or K562 cells affected by the interaction of LAL2, immunofluorescence assays were performed with LAL2 (labeled with Alexa 555) and LIP (labeled with Alexa 488). Thereafter, MCF-7 or K562 cells were incubated with LAL2 alone, LIP alone, or the combination of LAL2 and LIP. The results revealed that rLAL2 could bind to MCF-7 or K562 cells and was not affected by LIP. When Alexa 555-LAL2 and Alexa 488-LIP were added together to MCF-7/K562 cells, compared with Alexa 488-LIP treated alone, the number of cells located by Alexa 488-LIP increased. As the concentration of LAL2 increased, the number of cells located by Alexa 488-LIP gradually increased (Figures 4G,H).

### LAL2 Involved in the Immune Response of Bacteria and Poly I:C

**Figure 5:**
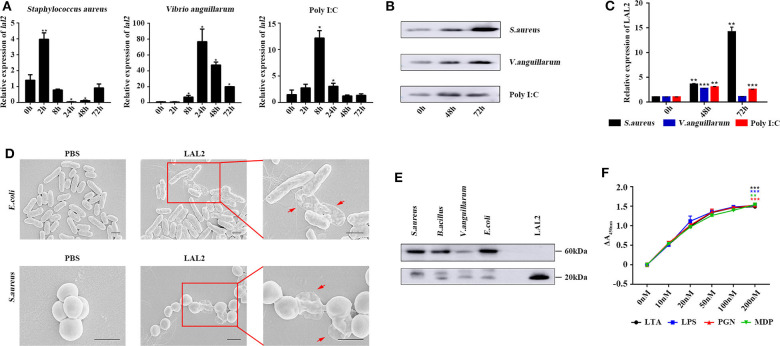
LAL2 plays an important role in the antibacterial and antiviral process. **(A)** Q-PCR was used to determine the relative expression of *lal2* genes after stimulating (*n* = 3). **(B)** After 48 and 72 h stimulation with different immunogens, western blot detected LAL2 protein expression level in lamprey serum (*n* = 3). **(C)** The summary graph of LAL2 relative expression by calculating the ratio of gray values. **(D)** Scanning electron microscope image of bacteria after LAL2 incubation. In the LAL2 treatment group, the right picture is an enlarged view of the left picture. Scale bar: 1 μm. **(E)** The binding of microorganisms by LAL2 proteins. Every living microbial strain was incubated with 10 μg natural LAL2 proteins in PBS buffer for 12 h, and the washed pellets were subjected to 15% SDS-PAGE and detected using western blot with an anti-LAL2 antibody. LAL2 protein 0.5 μg is used as positive control. **(F)** ELISA analysis of the interaction between LAL2 and microbial components (*n* = 3) *P < 0.05, **P < 0.01, ***P < 0.001.

Based on the above experimental results, it is speculated that LAL2 plays a role in the lamprey immune response. To verify this hypothesis, by means of Q-PCR, the temporal expression of *lal2* genes was detected after stimulating lamprey with gram-positive bacterium, gram-negative bacterium, or Poly I:C virus mimic for 0, 2, 8, 24, 48, or 72 h (Figure 5A). The results showed that the expression of *lal2* mRNA was up-regulated significantly (*P* = 0.0005) in the *S. aureus* stimulation group, and reached the maximal level at 2 h post-stimulation, which was 4.0-fold compared with the 0 h group. In the *V. anguillarum* stimulation group, the expression of *lal2* was strongly up-regulated (*P* = 0.0214), and reached the maximal level at 24 h, which was 76.8-fold compared with the blank group. The expression of *lal2* was also significantly increased (*P* = 0.0134) with Poly I:C stimulation and reached the maximal level at 8 h, which was 12.2-fold compared with the control group. In the case of the same amount of total serum protein, the protein levels of LAL2 were significantly increased by stimulation of *S. aureus, V. anguillarum*, and Poly I:C over 48 and 72 h (Figures 5B,C).

Subsequently, we used *S. aureus* and *E. coli* to detect the antibacterial activity of LAL2 (Figure 5D). Different bacterial strains were incubated with the purified natural LAL2 proteins in PBS as the experimental group (Figure S5), the control group was incubated with PBS. The morphological changes of the bacteria were observed using scanning electron microscopy. After LAL2 incubation, *S. aureus* and *E. coli* had changed in morphology, the cell surface appeared wrinkly and sunken, cell contents were released into the culture media, compared with the control group (areas indicated by red arrow). It was suspected that LAL2 could play a role in antibacterial activity by combining with bacteria to perform bacterial killing and clearance. Thereafter, four different bacterial strains were incubated with natural LAL2 proteins, including *S. aureus, B. cereus, V. anguillarum*, and *E. coli*, and the bacterial pellets were washed and analyzed using western blot with anti-LAL2 polyclonal antibody, indicating that LAL2 proteins could bind to gram-positive bacteria (*S. aureus* and *B. cereus*) and gram-negative bacteria (*E. coli*) in the form of a trimer. However, the combination with *V. anguillarum* was weak (Figure 5E). To explore the mechanism of combination, we used ELISA to evaluate the interaction between the natural LAL2 protein and different bacterial cell wall components (Figure 5F). The results showed that LAL2 could interact with soluble peptidoglycan (PGN), LTA, and LPS in a dose-dependent manner, and LAL2 could also bind specifically to minimal PGN motif muramyl dipeptide (MDP).

## Discussion

In 1986, M. Pontes et al. suggested that a similar amino acid composition exists for LrLAL1, LrLAL2, and MmAPOA4 (1). Later in 1996, Le Wang et al. performed a practical analysis of the systematic evolution of the apolipoprotein multigene family and found that the common ancestors of APOA1, APOA2, APOA4, and APOE may have appeared 460 million years ago in an ordovician vertebrate, which may be related to the major apolipoprotein LAL1 and LAL2 in Lamprey (30). Recently, Liu et al. postulated that the ancestral members of apolipoprotein are likely APOA1 and/or APOA4, and that other apolipoproteins emerged subsequently by gene duplication (31). However, our study can trace back to their common vertebrate ancestor, lamprey, indicating that LAL1 and LAL2 are indeed apolipoproteins. Furthermore, LAL2 is located in an evolutionary original position relative to LAL1, with motifs 4 and 5 in LAL2 obtained from amphioxus APOA1, and LAL1 formed by loss of motif 5, inversion, and insertion of the transposon into another scaffold (Figure 2E). We, therefore, postulate the following scenario: a series of duplication events beginning from *lal2* and *lal1* (Figure 2C), produced on zebrafish chromosome 16, resulting in the following apolipoprotein genes: *apoa1, apoa2*, and *apoe*. Subsequently, *apoa1* underwent tandem duplications and produced *apoa4*, while *apoc* is generated by *apoa1*/*apoa4* fragments. A DNA transposition then resulted in the insertion of the *apoc3* gene in between the *apoa1* and *apoa4* genes (28). The *apoa2* gene moved to human chromosome 1 and the genes for *apoa1, a4*, and *c3* moved to human chromosome 11. During species evolution, *apoc1, apoc2*, and *apoe* were lost in Aves. Eventually the other apolipoprotein genes, *apoe, c1, c2*, and *c4*, remained as a cluster on human chromosome 19. In the current study, it is speculated that the ancestral apolipoprotein gene may be subjected to different selection pressures at the same time during early differentiation. The order of evolution may be LAL2/LAL1—APOA1/APOA4/APOE—APOA2/APOC1/APOC2—APOC3/APOA5—APOC4 (28, 30–34).

Fitch et al. observed that human APOA1 contains multiple repeats of 22 amino acids (22-mer), each repeat is a tandem array of two 11-mers (28, 33). It is suggested that the repeat unit of 22-mer has been a structural element that builds an amphipathic α-helix. In addition, the existence of a 22-mer periodicity has also been found in other apolipoproteins, including APOA2, A4, C2, C3, and E, and an 11-mer has been found in APOC1. Lamprey LALl has a repeat pattern similar to those in human APOA1 and APOC3, while there is no clear indication for the presence of internal duplication in LAL2 sequence (28). Moreover, since there are a large number of α-helices in LAL1 and LAL2, the segments 79–99 and 100–120 of LAL2 have the potential to form an amphipathic helical structure. The hydrophilic residues on one side of the amphipathic helix keep the apolipoproteins at the surface of the lipoprotein particle to facilitate transfer between lipoprotein particles and interaction with other molecules, such as enzymes and specific cell surface receptors. New motifs are gradually evolved in LAL1 and LAL2 to form the apolipoprotein functional domain and conservative α-helix to ensure better survival, thus producing several types of apolipoproteins with significantly different structures.

Both the mRNA and protein levels of LAL2 were primarily expressed in liver, leukocytes, and kidney of the lamprey, which differed from the tissue distribution observed for apolipoprotein expression in teleost fish. For example, most apolipoprotein genes exhibited tissue-specific expression patterns in intestine, liver, and skin of channel catfish (35). Moreover, human *apoa1, apoa4*, and *apoc3* have been cloned in fetal intestine and adult liver but not in fetal liver, kidney, heart, brain, or muscle (36). Hence, with evolution, the distribution of apolipoproteins has gradually become regionalized to further perform unique functions. However, liver, leukocytes, and kidney are important immune tissues, indicating that LAL2 is likely to play a critical role in immune defense. In fact, our previous study demonstrated that LIP is primarily distributed in the supraneural body and leukocytes of the lamprey (21), while LAL2 is abundant in the sera (Figure 1A, indicated by the red arrow), suggesting that LAL2 protein acts as a secreted protein and participates extensively in blood circulation to accomplish immune responses. Additionally, the unique recognition mechanism of LIP is dependent on binding with both N-linked glycans on GPI-Aps, and SM in lipid rafts (29). We, therefore, expect that LAL2 can assist LIP in the diagnosis and control of tumor cells via targeted human cancer therapies.

Comprehensive functional analyses revealed the role of lamprey LAL2 and immune responses (Figure 5). Insect apolipoproteins were shown to cooperate against pathogens, such as silkworm apolipophorins (13, 37–39). In respect with the immune defense, LAL2 exerts unique biological functions in synergy with LIP. Moreover, *lal2* was up-regulated after stimulating lamprey with gram-positive bacteria, gram-negative bacteria, and Poly I:C virus mimic, respectively. Scanning electron microscope (SEM) images show that LAL2 can destroy the structure of *S. aureus* and *E. coli* and influence bactericidal activity. This is similar to the results highlighting that high-density lipoprotein (HDL) in the carp epidermis is secreted into mucus and performs antibacterial activity (40). This lipoprotein is mainly composed of two major apolipoproteins (APOA1 and APOA2), which correspond to the most abundant plasma proteins in several bony fish and have antibacterial activity. Orange-spotted grouper *E. coioides* APOA1 can inhibit the replication of Singapore grouper iridovirus (SGIV), and up-regulate the expression of its immune-related genes, ISG15 and Mx-I (9).

Microbes express signature molecules known as pathogen-associated molecular patterns (PAMPs), such as LTA, PGN, and MDP in gram-positive bacteria, and LPS in gram-negative bacteria (41). Wang et al. found that *Branchiostoma belcheri* rAPOA1 can bind LPS and LTA of various gram-positive and gram-negative bacteria and exhibits antibacterial activity against gram-negative bacteria *in vitro* (42). Silkworm apolipophorin protein inhibits hemolysin gene expression of *S. aureus* via binding to cell surface LTA (13). Conserved high amphipathic α-helical content between fish and mammal apolipoproteins can neutralize LPS via the CD14/TLR4 (Toll Like Receptor 4) pathway and intercalate into lipid bilayers to resist bacterial invasion (16, 43, 44). To investigate the antibacterial mechanism of lamprey LAL2, ELISA results show LAL2 can bind to LPS, LTA, PGN, and MDP due to highly homologous α-helical content with human APOA1 (Figure S4). In fact, APO-II/I proteins may either shuttle APO-III and other immune proteins to microbial surfaces, contribute to microbial clearance, or detoxify immune-stimulatory cell wall components (40). The lipid particles nucleated by lipid carrier proteins in the hemolymph may serve as platforms for recruiting immunity proteins (45). Future studies are required to elucidate the interaction between molecules in sera with LAL2 and the signaling pathway involving LAL2 in order to further unravel immune defense in lampreys.

In conclusion, this study identified the molecular evolution and tissue distribution of lamprey LAL2. Furthermore, we demonstrate that lamprey LAL2 can serve as an effector molecule in sera for immune responses, pattern recognition, and bactericidal activity. Our studies not only help to expand on the evolutionary history of the vertebrate apolipoprotein multigene family, but also provide new insight into the important and diversified functional properties of apolipoprotein.

## Data Availability Statement

Data can be found on Genbank—MN893307 and MN893306. Other raw data supporting the conclusions of this article will be made available by the authors, without undue reservation, to any qualified researcher.

## Ethics Statement

The animal experiments were performed in accordance with the regulations of the Animal Welfare and Research Ethics Committee of the Institute of Dalian Medical University's Animal Care protocol (Permit Number: SCXK2008-0002).

## Author Contributions

QL, YP, and QH designed the experiments. Flow cytometry and immunohistochemistry were finished by QH. HW, QH, and YH analyzed the experimental results. QH and YP wrote the manuscript. All authors contributed to the article and approved the submitted version.

## Conflict of Interest

The authors declare that the research was conducted in the absence of any commercial or financial relationships that could be construed as a potential conflict of interest.

## Supplementary Material

Figure S1 Ten amino acid test maps of LAL2 N-terminal sequence (number 2).

Figure S2 Sequence alignment results of LAL2 in *Lampetra japonica, Petromyzon marinus*, and *Lampetra fluviatilis*.

Figure S3 The secondary structure of LAL2 and LAL1. **(A)** The predicted results on PSIPRED website of LAL2 secondary structures. Alpha helix, extended strand, beta turn, and random coil account for 78.01, 5.76, 3.14, and 13.09%, respectively. **(B)** The predicted results on PSIPRED website of LAL2 secondary structures. Alpha helix, beta turn, and random coil account for 79.05, 5.71, and 15.24%, respectively. **(C)** Circular dichroism shows two negative peaks and one positive peak in LAL2, which conform to the CD spectrum of the α-helix in the secondary structure of the protein (negative peaks at 208 and 222 nm, and positive peaks near 190 nm). **(D)** Sequence alignment analysis of sequence similarity between LrLAL2 and HsAPOA1. LAL2 is highly similar to the APOA1 sequence Helix3 (88–98), and it is also similar to the APOA1 sequence Helix4, 5, and 6 (99–120, 121–142, 143–164).

Figure S4 Expression and purification of rLAL2 protein and preparation of antibodies. **(A)** Prokaryotic expression and purification of rLAL2. M, low molecular-weight protein marker; *lane 1*, non-induced Rosetta/pET32a-*lal2* cells; *lane 2*, induced Rosetta/pET32a-*lal2* cells using IPTG; *lane 3*, purified LAL2 recombinant protein, black line points at the target protein. **(B)** ELISA assay to assess the serum anti-LAL2 polyclonal antibody titer from two rabbits. **(C)** Analysis of the specificity of the rabbit anti-LAL2 polyclonal antibody using western blot. *lane 1*, LAL2 recombinant protein; *lane 2*, lamprey serum. **(D)** LC-MS/MS analysis trypic-digested peptides of LAL2-Dimer.

Figure S5 Purification of natural LAL2 protein with anion exchange chromatography. (Left) The map of natural LAL2 protein purification process with anion exchange chromatography from UNICORN 7.0; (Right) Purification of natural LAL2 protein. M, low molecular-weight protein marker; lane 1, No. 46 serum sample; lane 2, Flow-through fluid; lane 3, Equilibrium fluid; lane 4, Eluent 20; lane 5, Eluent 25; lane 6, Eluent 30.
