# Supplementary material for: Molecular Evolution of Apolipoprotein Multigene Family and the Original Functional Properties of Serum Apolipoprotein (LAL2) in Lampetra japonica
Source: Front Immunol. 2020 Aug 11;11:1751. doi: 10.3389/fimmu.2020.01751 (PMC7431520; doi:10.3389/fimmu.2020.01751)
Supplement: Supplementary file 1 [file Table_1.DOC]

*Table S1. LC/MS/MS analysis of tryptic-digested peptides of LAL2-1*

| **m/z meas.** | **Score** | **Sequence** |
| --- | --- | --- |
| 548.2816 | 63.9 | K.TYLETALER.L |
| 631.7934 | 60.9 | R.LHSYGEAVSGDK.A |
| 736.3385 | 18.4 | R.LHSYGEAVSGDKADGIMTEAR.E |
| 482.2311 | 73.1 | K.ADGIMTEAR.E |
| 490.2289 | 53.0 | K.ADGIMTEAR.E |
| 526.7860 | 76.3 | K.ALPEGVTTHK.L |
| 632.2848 | 46.8 | K.LAEEMAEAANAK.L |
| 624.2914 | 72.9 | K.LAEEMAEAANAK.L |
| 476.8126 | 45.5 | K.LVPILQAAK.A |
| 764.9290 | 58.1 | R.VTAHLHESAPLIIK.V |
| 418.7367 | 26.6 | R.GFIESKR.G |
| 725.8658 | 72.2 | R.GVMWAYLAALAER.A |
| 733.8604 | 85.5 | R.GVMWAYLAALAER.A |
| 452.2615 | 30.7 | K.AKLDDTLK.G |

*Table S2. LC/MS/MS analysis of tryptic-digested peptides of LAL2-2*

| **m/z meas.** | **Score** | **Sequence** |
| --- | --- | --- |
| 548.2792 | 63.9 | K.TYLETALER.L |
| 631.7946 | 58.7 | R.LHSYGEAVSGDK.A |
| 482.2298 | 73.2 | K.ADGIMTEAR.E |
| 490.2272 | 47.7 | K.ADGIMTEAR.E |
| 526.7862 | 76.9 | K.ALPEGVTTHK.L |
| 632.2876 | 64.4 | K.LAEEMAEAANAK.L |
| 624.2894 | 79.7 | K.LAEEMAEAANAK.L |
| 476.8118 | 43.8 | K.LVPILQAAK.A |
| 764.9268 | 57.0 | R.VTAHLHESAPLIIK.V |
| 418.7346 | 39.5 | R.GFIESKR.G |
| 803.9097 | 63.0 | K.RGVMWAYLAALAER.A |
| 541.6146 | 58.8 | K.RGVMWAYLAALAER.A |
| 725.8615 | 68.8 | R.GVMWAYLAALAER.A |
| 733.8611 | 85.1 | R.GVMWAYLAALAER.A |
| 452.2606 | 29.0 | K.AKLDDTLK.G |

*Table S3. Best possible amino acid sequence of motifs.*

| Motif number | Best possible match | Width |
| --- | --- | --- |
| 1 | MKAVVLTLALLFLTG | 15 |
| 2 | VSWEQVKBAFWDYVS | 15 |
| 3 | QLTTTAEEALEEJQKSZLTQZLRALISDT | 28 |
| 4 | YLZDLQZQLKPYAEELREQLS | 21 |
| 5 | ELKQKLAPYAEGLRTSIZENLDQLRERLM | 29 |
| 6 | RLEQNVEELKGRLTPYAEELRARJGZHVEELRRSLAPYAEELRERLSRSV | 50 |
| 7 | FRRKVEPYIEEFTKALQQZAEZLRQKLEP | 29 |
| 8 | PPPGHSAFAPEFGQSDSGKVLSKLQARLDDLWEDIAHGLQDQGHSHLGDP | 50 |
| 9 | IRLQAEIFQARLKGWFEPJVEDMQRQWAGLMEKIQAAVGTNPIPVP | 46 |
