# Supplementary figures and images for: Molecular Evolution of Apolipoprotein Multigene Family and the Original Functional Properties of Serum Apolipoprotein (LAL2) in Lampetra japonica
Source: Front Immunol. 2020 Aug 11;11:1751. doi: 10.3389/fimmu.2020.01751 (PMC7431520; doi:10.3389/fimmu.2020.01751)

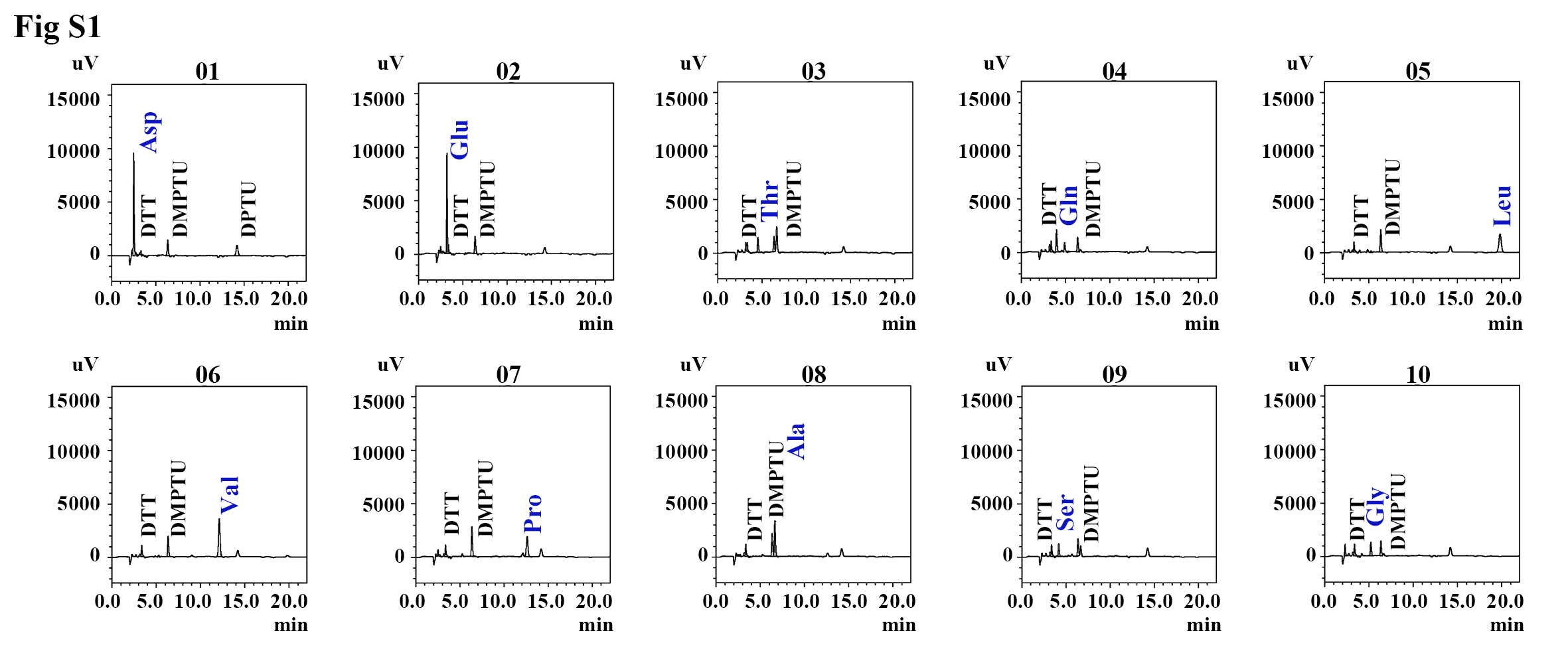

Supplement: Figure S1 — Ten amino acid test maps of LAL2 N-terminal sequence (number 2). [file Image_1.JPEG]

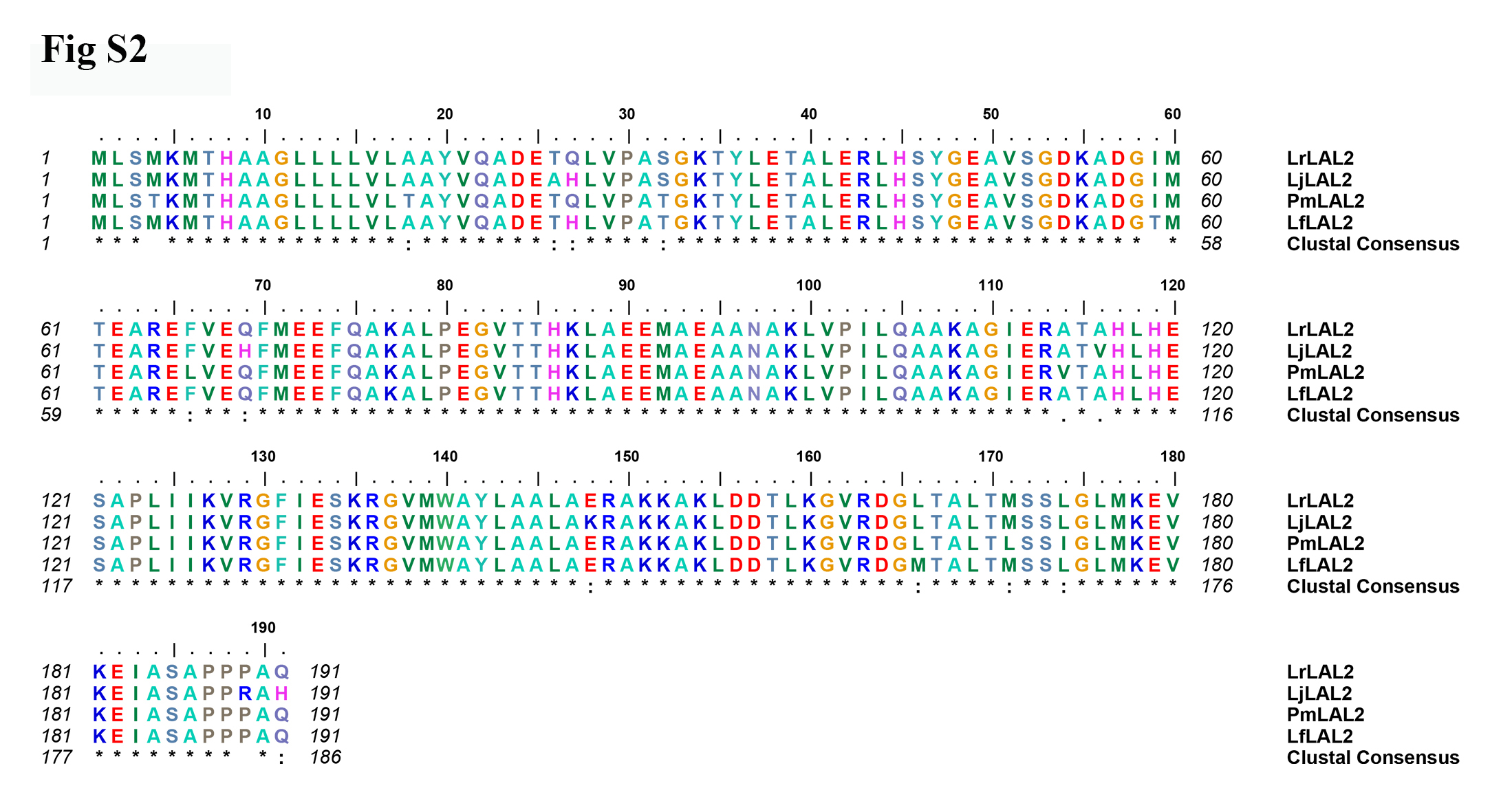

Supplement: Figure S2 — Sequence alignment results of LAL2 in Lampetra japonica, Petromyzon marinus, and Lampetra fluviatilis. [file Image_2.JPEG]

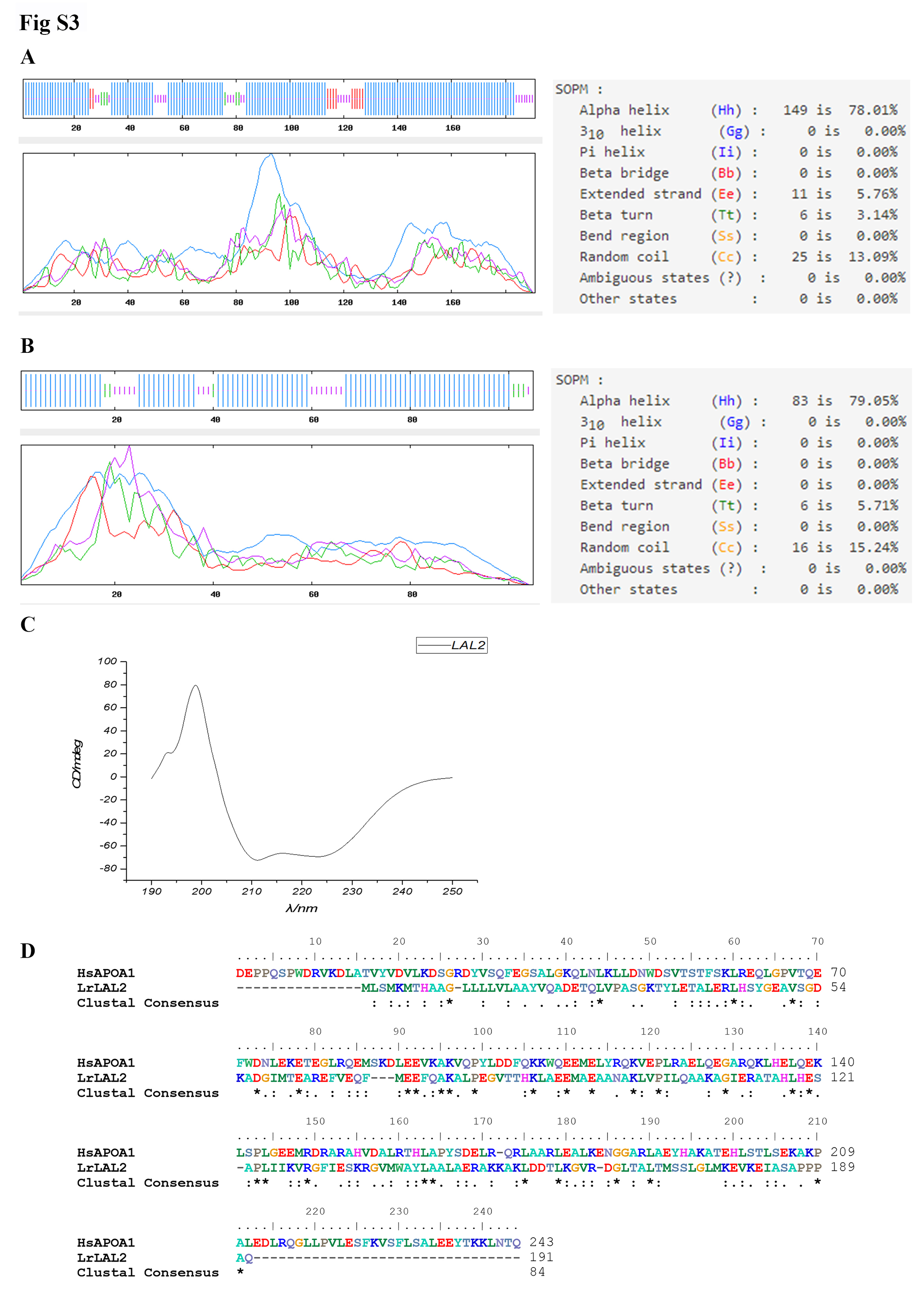

Supplement: Figure S3 — The secondary structure of LAL2 and LAL1. (A) The predicted results on PSIPRED website of LAL2 secondary structures. Alpha helix, extended strand, beta turn, and random coil account for 78.01, 5.76, 3.14, and 13.09%, respectively. (B) The predicted results on PSIPRED website of LAL2 secondary structures. Alpha helix, beta turn, and random coil account for 79.05, 5.71, and 15.24%, respectively. (C) Circular dichroism shows two negative peaks and one positive peak in LAL2, which conform to the CD spectrum of the α-helix in the secondary structure of the protein (negative peaks at 208 and 222 nm, and positive peaks near 190 nm). (D) Sequence alignment analysis of sequence similarity between LrLAL2 and HsAPOA1. LAL2 is highly similar to the APOA1 sequence Helix3 (88–98), and it is also similar to the APOA1 sequence Helix4, 5, and 6 (99–120, 121–142, 143–164). [file Image_3.jpg]

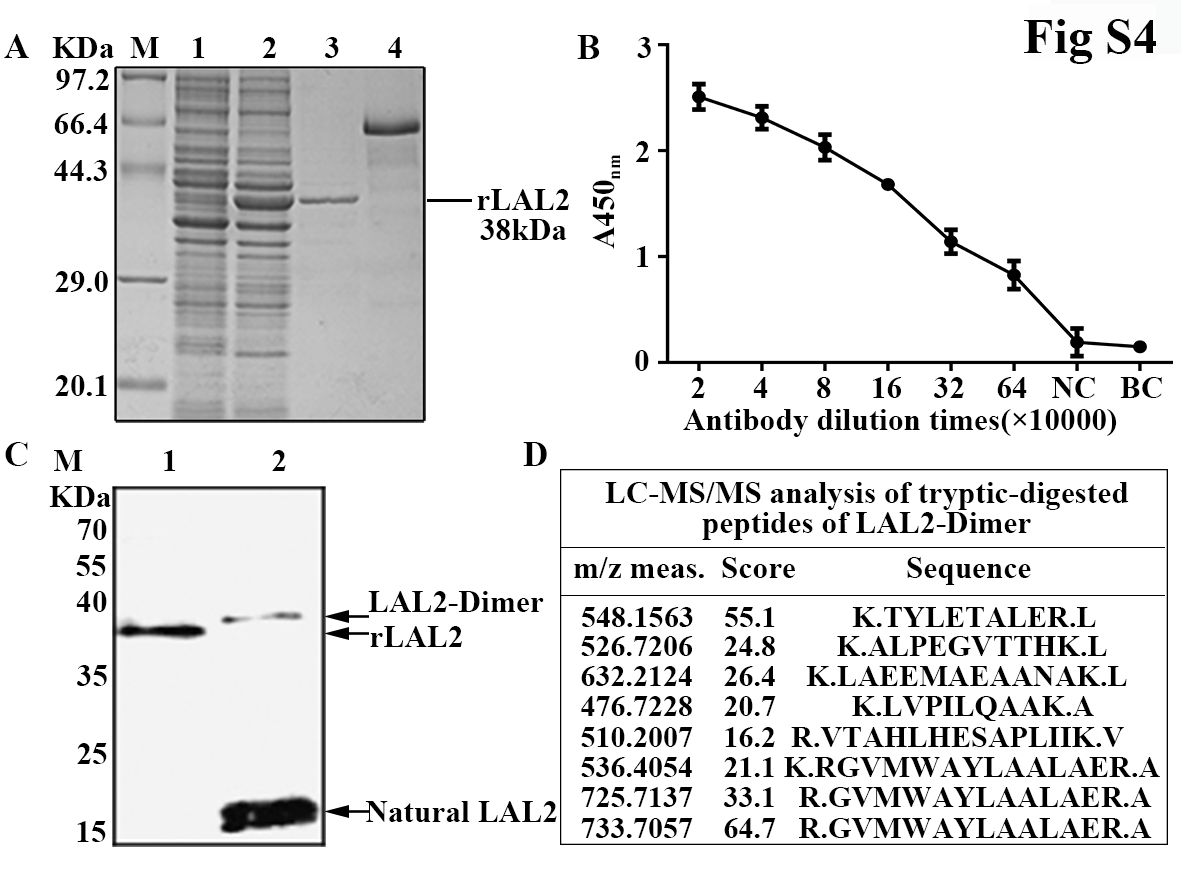

Supplement: Figure S4 — Expression and purification of rLAL2 protein and preparation of antibodies. (A) Prokaryotic expression and purification of rLAL2. M, low molecular-weight protein marker; lane 1, non-induced Rosetta/pET32a-lal2 cells; lane 2, induced Rosetta/pET32a-lal2 cells using IPTG; lane 3, purified LAL2 recombinant protein, black line points at the target protein. (B) ELISA assay to assess the serum anti-LAL2 polyclonal antibody titer from two rabbits. (C) Analysis of the specificity of the rabbit anti-LAL2 polyclonal antibody using western blot. lane 1, LAL2 recombinant protein; lane 2, lamprey serum. (D) LC-MS/MS analysis trypic-digested peptides of LAL2-Dimer. [file Image_4.tif]

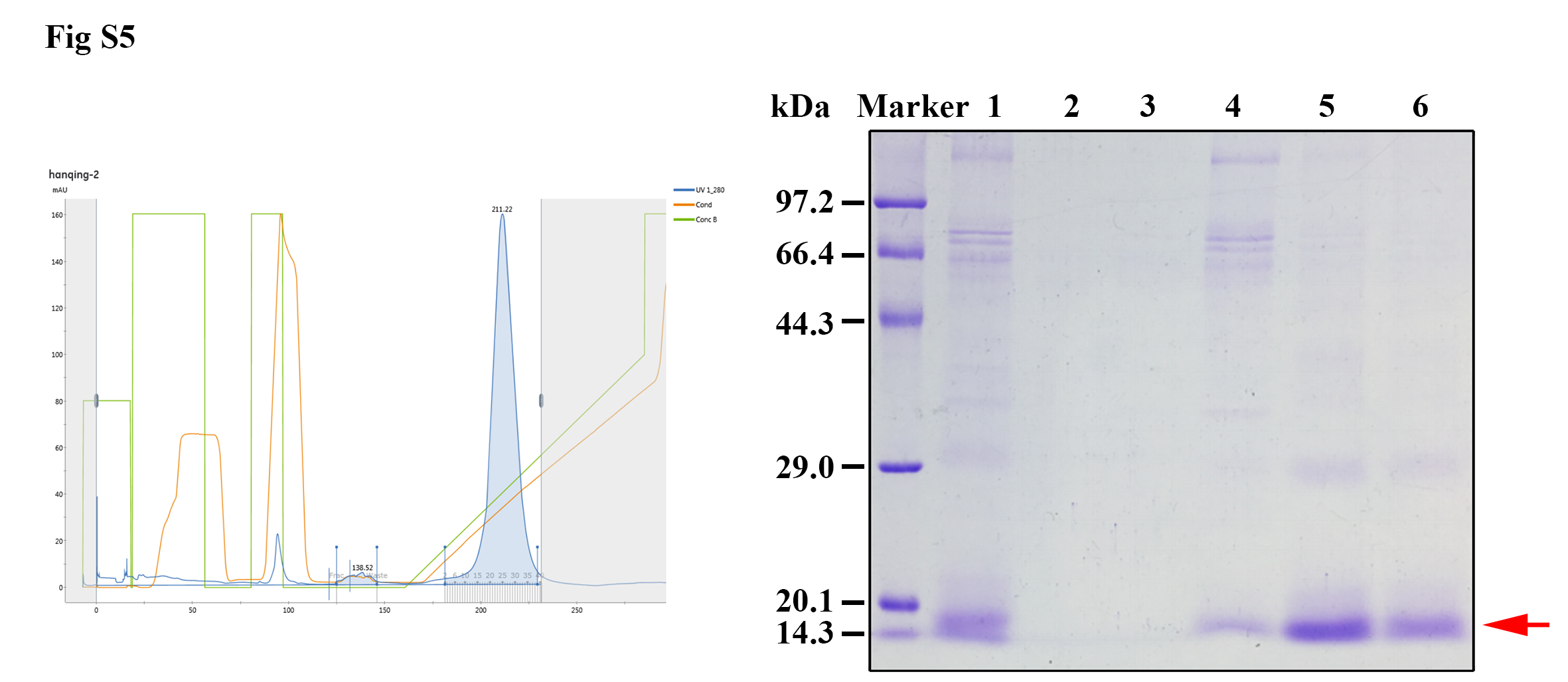

Supplement: Figure S5 — Purification of natural LAL2 protein with anion exchange chromatography. (Left) The map of natural LAL2 protein purification process with anion exchange chromatography from UNICORN 7.0; (Right) Purification of natural LAL2 protein. M, low molecular-weight protein marker; lane 1, No. 46 serum sample; lane 2, Flow-through fluid; lane 3, Equilibrium fluid; lane 4, Eluent 20; lane 5, Eluent 25; lane 6, Eluent 30. [file Image_5.tif]
